# The impact of disease activity and interferon-α on the nervous system in systemic lupus erythematosus

**DOI:** 10.1186/s13075-025-03539-2

**Published:** 2025-03-20

**Authors:** Kristoffer A. Zervides, Elsa Grenmyr, Shorena Janelidze, Petrus Linge, Jessika Nystedt, Petra C. Nilsson, Pia C. Sundgren, Oskar Hansson, Anders A. Bengtsson, Andreas Jönsen

**Affiliations:** 1 Department of Clinical Sciences, Rheumatology, Lund University, Skåne University Hospital, Lund, Sweden; 2 Department of Clinical Sciences, Neurology, Lund University, Skåne University Hospital, Lund, Sweden; 3 Clinical Memory Research Unit, Department of Clinical Sciences Malmö, Lund University, Lund, Sweden; 4 Department of Clinical Sciences, Diagnostic Radiology, Lund University, Skåne University Hospital, Lund, Sweden; 5 Lund University BioImaging Center, Lund University, Lund, Sweden; 6 Memory Clinic, Skåne University Hospital, Malmö, Sweden; 7 Skåne University Hospital, Neurology Clinic, Entrégatan 7, Lund, 221 85 Sweden

**Keywords:** Systemic lupus erythematosus, Disease activity, Interferon, Neurofilament, MRI, Cognitive dysfunction

## Abstract

**Background:**

Systemic lupus erythematosus (SLE) patients, with or without neuropsychiatric SLE (NPSLE), exhibit greater neuronal impairment compared to healthy individuals in terms of neuronal damage, magnet resonance imaging (MRI) changes and cognitive dysfunction. Interferon (IFN)-α is a key immunopathogenic driver of SLE, being persistently overexpressed in the majority of patients. This longitudinal study aimed to investigate whether disease activity and serum IFN-α levels over time were associated with objective findings of neuronal impairment regarding (i) higher plasma neurofilament light (NfL) concentrations, (ii) structural alterations on MRI, and (iii) cognitive dysfunction upon testing.

**Methods:**

Sixty-six consecutive female SLE outpatients were enrolled in a cross-sectional study. Retrospectively, prior visits with concomitant blood samples (*n* = 199) were selected from the Lund Lupus Cohort database and biobank. Serum IFN-α concentrations were measured using an electrochemiluminescence immunoassay. IFN-α lupus phenotypes were defined as high (*n* = 24) or low (*n* = 33) by considering persistent elevations in serum IFN-α concentrations despite low SLE Disease Activity Index-2000 (SLEDAI-2 K) scores. SLEDAI-2 K lupus phenotypes were defined as moderate-high (*n* = 31) or low (*n* = 35) based on SLEDAI-2 K scores from all 576 available visits prior to the study. Ongoing neuronal damage was assessed by plasma NfL concentration measurements using Simoa at the 199 visits. Structural MRI alterations and cognitive dysfunction according to the CNS-Vital Signs test battery were the additional outcomes. Multivariate linear mixed-effect, linear regression, and logistic regression models were used for the statistical analyses.

**Results:**

Visits with higher disease activity were associated with higher plasma NfL concentrations (e.g. SLEDAI-2 K total: *p* = 1.5*10^− 6^). High compared with low IFN-α lupus phenotype patients displayed more cognitive dysfunction (odds ratio 11.0, *p* = 0.004), and smaller volumes of total grey matter, caudate nucleus, and thalamus (*p* = 0.036; *p* = 0.038; *p* = 0.023). Moderate-high compared with low SLEDAI-2 K lupus phenotype patients displayed larger white matter lesion volumes and smaller total grey matter and thalamus volumes (*p* = 0.011; *p* = 0.041; *p* = 0.005).

**Conclusions:**

The study suggests that disease activity and IFN-α may drive neuronal affliction in SLE, also in the absence of overt neuropsychiatric symptoms, and that controlling disease activity could improve the cerebral outcome.

## Background

Systemic lupus erythematosus (SLE) is a relapsing-remitting systemic disease with heterogeneous presentations predominantly affecting young females [1]. The complex pathogenesis of SLE involves breakdown of tolerance to nucleic acids, cytosolic-, cell surface-, and extracellular products, activation of the complement system, formation of immune complexes, and activation of the type 1 interferon (IFN) system [2]. In particular, IFN-α plays a pivotal role in the SLE pathogenesis, being chronically and persistently overexpressed in 50–70% of SLE patients, despite treatment and changes in disease activity [3, 4]. A positive IFN-α signature corresponds to a greater propensity to severe manifestations and increased disease activity [3].

Twelve to 95% of patients experience clinical manifestations from the nervous system, neuropsychiatric SLE (NPSLE) [5]. The wide-spread frequency is mainly due to methodological differences; some NPSLE attribution models are more stringent, excluding non-specific manifestations prevalent in the background population such as mood disorders, cognitive dysfunction, and headache [6–9]. Overt NPSLE manifestations, such as demyelinating disease, myelopathy, aseptic meningitis, and stroke, are less common and easier to identify as attributable to SLE [8, 10]. Conversely, cognitive dysfunction, fatigue, and mood disorders, symptoms common in patients with and without NPSLE, remain poorly understood from a biological perspective, and have substantial negative effects on function and quality of life [11]. According to magnetic resonance imaging (MRI) studies from us and others, the brains of persons with SLE are altered compared with healthy controls, regardless of neuropsychiatric involvement according to the established NPSLE attribution models, and consistently, neuroimaging and laboratory biomarkers may not always differentiate between SLE patients with and without NPSLE [10, 12–16].

The pathogenesis of nervous system involvement can be depicted along two overlapping pathways [10, 13]. The inflammatory pathway involves blood-brain barrier dysfunction, microglial activation, and brain-reactive autoantibodies, among other factors. The ischemic pathway is facilitated by endothelial dysfunction, cerebral microangiopathy, and subsequent thrombosis, and may be influenced by factors from the inflammatory pathway, such as general SLE-inflammation and antiphospholipid antibodies. Although IFN-α has been unsuccessfully assessed as a diagnostic biomarker to discern patients with and without NPSLE, the consequences on the brain of persistently elevated IFN-α have not been assessed in SLE [17]. In genetic interferonopathies, centrally produced IFN-α target endothelial cells resulting in neurotoxic effects mediated by cerebral microangiopathy, and consequently, neurodegeneration arises [18]. Patients undergoing systemic IFN-α therapy for other disorders frequently develop cognitive impairment, which may be a consequence of impaired whole brain functional connectivity and efficiency [19–21].

Neurofilament light (NfL) is a protein component of the neuronal cytoskeleton which is released into the cerebrospinal fluid (CSF) and plasma upon neuronal damage in response to normal ageing, and to a larger extent under neuropathological circumstances including neurodegenerative and neuroinflammatory conditions such as acute NPSLE [22–25]. NfL may serve as an indicator of subclinical neuronal damage in subjects without primary neurological disorders and as a predictor of cognitive decline in patients with and without neurodegenerative disorders [26–29]. Cross-sectionally, we and others demonstrated higher plasma NfL concentrations in SLE patients with or without NPSLE compared with matched controls, further supporting that neuronal damage is increased in SLE regardless of NPSLE [16, 30]. Due to the cross-sectional nature of these studies, it remained unknown when in the disease course increased neuronal damage occurs and whether it is persistently elevated or fluctuates concomitantly with disease activity, as it has been observed with structural brain atrophy on MRI [31]. However, particularly high NfL levels were displayed in patients with a more severe lupus phenotype, indicating a higher degree of neuronal damage in patients with a higher cumulative disease burden.

These findings lead us to the hypothesis that pivotal immunopathogenic disease drivers in SLE, such as IFN-α, and the overall SLE inflammatory activity may drive the accelerated neuronal damage observed in SLE patients, with or without NPSLE, ultimately leading to MRI alterations and cognitive dysfunction. The aim of this longitudinal study is to explore whether the overall SLE disease activity and serum IFN-α levels over time are associated with negative effects on the brain regarding (i) higher plasma NfL concentrations as an indicator of increased neuronal damage, (ii) structural alterations on MRI, and (iii) cognitive dysfunction upon testing. Thus, objective findings of neuronal affliction will serve as outcomes, rather than clinical manifestations, as these findings are altered in SLE patients regardless of the presence or absence of NPSLE.

## Methods

### Study participants and visits at the department of rheumatology

**Fig. 1:**
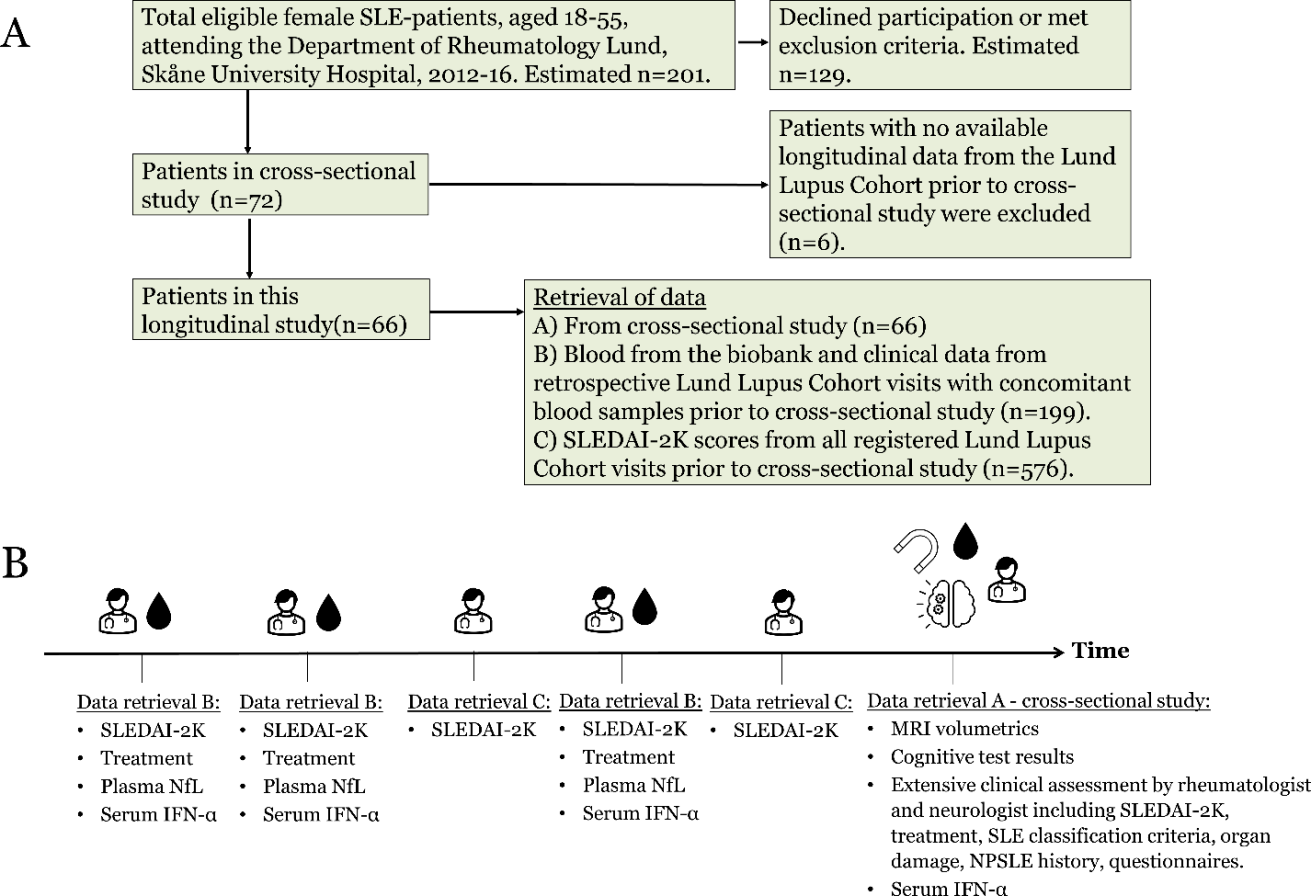
Study design and data retrieval. **A** This flow-chart depicts the overall study design including the recruitment of patients for the cross-sectional study and the retrieval of retrospective longitudinal data from the Lund Lupus Cohort database and biobank. **B** The timeline illustrates the data retrieval for the study. Data retrieval A: In the cross-sectional study patients were assessed extensively by a rheumatologist, neurologist, underwent cognitive testing by a neuropsychologist, MRI, and blood samples. This data was used to assess the associations between the lupus phenotypes and MRI changes & cognitive dysfunction. Data retrieval B: Retrospectively, we chose 199 visits with concomitant blood samples with high and low disease activity, including one visit as early as possible. This data was used for investigating the SLEDAI-2 K and IFN-α associations with repeatedly measured plasma NfL, and to collect samples to determine the IFN-α lupus phenotypes. Data retrieval C: All 576 prior visits with SLEDAI-2 K registered in the database were retrieved. This data was used to determine the SLEDAI-2 K lupus phenotypes

At the Department of Rheumatology in Lund, Skåne University Hospital, Sweden, all SLE patients are asked to prospectively supply clinical information to our research database for research purposes (Fig. 1A) [32]. The vast majority of visits from the resulting Lund Lupus Cohort are outpatient visits, and typically data and blood samples from hospitalisations in other departments are not obtained. Blood samples obtained at visits are stored at -80⁰ Celsius in our biobank.

### Cross-sectional data collection

All female SLE patients aged 18–55 attending the tertiary outpatient clinic during 2012 till early 2016, were asked consecutively to participate in a cross-sectional study, independently of disease activity and NP symptoms (Fig. 1A) [16, 33]. Exclusion criteria were any contraindication to MRI, left-handedness, pregnancy, and prior diagnosis with demyelinating disease, amyotrophic lateral sclerosis, or dementia. All patients fulfilled the Systemic Lupus Erythematosus International Collaborating Clinics (SLICC) Classification Criteria for SLE [34]. By not including male sex and older patients we aimed to reduce the study group heterogeneity and age-related cognitive decline and MRI abnormalities. Based on the number of SLE patients attending our outpatient clinic at the time of the study, we estimate that 201 patients were eligible to participate in this study (Fig. 1A). Detailed non-participation analysis could not be performed due to the lack of consent, however, aggregate demographic data of the eligible patients indicate that the median age, disease duration and prevalence of lupus nephritis did not significantly differ from those who did and did not participate (*p* = 0.14, *p* = 0.79, and *p* = 0.48, respectively). Of the 72 patients who participated in the cross-sectional study, six were excluded in this longitudinal study due to missing retrospective longitudinal data in the Lund Lupus Cohort database, resulting in a total of 66 patients (Fig. 1A).

SLE manifestations according to the SLICC Classification Criteria for SLE and organ damage according to the SLICC/American College of Rheumatology (ACR)-Damage Index (SDI) were retrieved from the Lund Lupus Cohort database from the time of the cross-sectional assessment [35] (Fig. 1B). A neurologist and a rheumatologist assessed all subjects for a history of NP-symptoms attributed by SLE according to a standardized protocol to define NPSLE according to three attribution models: the most stringent SLICC A and B models and the less stringent ACR model [8, 9]. The individual NPSLE manifestations are depicted elsewhere [33]. Questionnaires were used to evaluate smoking history, alcohol consumption, fatigue according to the Fatigue Severity Scale and Visual Analogue Scale 100 mm (VAS), and anxiety score and depression scores according to the Hospital Anxiety and Depression Scale (HADS) and Montgomery-Asberg Depression Rating Scale Self-rating version (MARDS-S) [33].

Sixty-five of the 66 subjects underwent neurocognitive testing by a neuropsychologist using the CNS Vital Signs (CNS-VS), a standardized test battery based on an age-matched normative comparison database [36]. The protocol consists of seven established tests, computing age-matched scores in 12 BRIEF-CORE Clinical Domains, five multiple test domains and seven single test domains, described in detail in a previous study [37]. Moderate to severe cognitive dysfunction in each domain was defined as Z-scores ≤ -1.4 of the age-matched standard score. Mild cognitive dysfunction was defined as Z-scores between − 1 and − 1.4.

All subjects, except one who could not complete the investigation, underwent an MRI scan at Lund University Bioimaging Center with the 3 Tesla Magnetom, Skyra, Siemens, Erlangen, Germany. The imaging protocol and post-processing analysis have been described in detail in another paper [38]. Analysis of regional brain volumes was performed by the semi-automatic cortical and subcortical segmentation software FreeSurfer version 5.3 (recon-all, with additional flags, -3T and MPRAGE). Analysis of white matter lesions (WML) was performed by segmenting data semi-automatically by the lesion growth algorithm using the Lesion Segmentation Toolbox version 2.0.14 for SPM12. Six subjects could not be properly evaluated for WML due to technical issues or motion artefacts. In this study, we used the following volumetric outcomes: volumes of WML, total white matter, total grey matter, total cortex, hippocampus, nucleus accumbens, amygdala, caudatus, globus pallidus, putamen, thalamus, corpus callosum, and total CSF spaces. The WML volumes were log10 (log)-transformed to obtain a normal distribution. All MRI variables were standardized according to estimated intracranial volume and were reported elsewhere [16].

### Longitudinal data collection

Longitudinally, clinical information and blood samples from the Lund Lupus Cohort database and biobank were collected from multiple retrospective visits from the 66 patients (Fig. 1). The Lund Lupus Cohort includes data from visits with prospectively scored disease activity, registration of medication and organ damage. We selected 199 visits with concomitant blood samples (median 3 visits and samples per patient), aiming to include visits with both high and low disease activity according to the SLE Disease Activity Index 2000 (SLEDAI-2 K), to include visits at evenly distributed time intervals for each patient, and to obtain data as soon as possible after SLE disease-onset, which was not possible for all patients (Fig. 1B) [39]. The information retrieved included year of diagnosis, SLEDAI-2 K scores, plasma creatinine concentrations, and ongoing treatment with antihypertensives, anticoagulation, antiplatelets, glucocorticoids, and disease-modifying antirheumatic drugs (DMARDs). We did not have information regarding co-morbid conditions over time. Active renal involvement was defined by the presence of SLEDAI-2 K points for at least one of the following items: hematuria, proteinuria, pyuria, or heme-granular/red blood cell urinary casts at the visit. Active skin or mucosal involvement was defined by the presence of SLEDAI-2 K points for at least one of the following items: inflammatory-type rash, alopecia, or mucosal ulcers at the visit.

In addition, we retrieved all available SLEDAI-2 K scores from the Lund Lupus Cohort database, from visits prior to the cross-sectional assessment, with the aim to define the disease activity lupus phenotype of each patient. If more than one visit were registered the same year, the visit with the highest SLEDAI-2 K score was selected. In total, 576 yearly visits with SLEDAI-2 K were included from the 66 patients (Fig. 1B).

### Laboratory analysis

All blood samples were processed using a standardized protocol. Plasma NfL concentrations were measurable in 196 of the 199 samples using a single-molecule array (Simoa; Quanterix; Billerica, MA) and the commercially available NfL assay was utilized (NF-light™ # 103186) (Fig. 1B). Plasma NfL was analysed in singlicates because previous studies using duplicates showed low intra-assay coefficient of variance [16]. Serum IFN-α concentrations were measured in 265 samples (66 from the cross-sectional timepoint together with the 199 retrospectively selected visits) using an electrochemiluminescence immunoassay (Meso Scale Diagnostics, S-Plex Human IFN-α2a Kit # K151P3S) (Fig. 1B). Reanalysis of selected serum samples confirmed assay-to-assay consistency (*r* = 0.90, *p* < 0.0001). Moreover, consistency across different methods was confirmed with strong concordance (*r* = 0.77, *p* < 0.0001) by comparing serum IFN-α concentrations obtained using the electrochemiluminescence immunoassay with those from quantitative polymerase chain reaction (qPCR) analysis in the selected samples. Plasma NfL and serum IFN-α concentrations were log-transformed to achieve a normal distribution.

### Defining lupus phenotypes

Patients were classified as having a moderate-high SLEDAI-2 K lupus phenotype if they exhibited SLEDAI-2 K ≥ 4 in at least 25% of their visits (*n* = 31) based on the 576 yearly visits (Fig. 1B). Patients were classified as having a low SLEDAI-2 K lupus phenotype if they displayed SLEDAI-2 K ≥ 4 in less than 25% of their visits (*n* = 35).

IFN-α lupus phenotypes were defined by assessing serum IFN-α concentrations during low disease activity visits (SLEDAI-2 K < 4) (Fig. 1B). If the majority of a patient’s low disease activity visits displayed serum IFN-α concentrations above or below the median IFN-α concentration (102 fg/ml), the individual was classified as having a high or low IFN-α lupus phenotype, respectively. If exactly half the registered IFN-α concentrations during low disease activity were above and half were below the median, the IFN-α lupus phenotype could not be defined (*n* = 2). Additionally, if a patient did not have any low disease activity visits registered, the IFN-α lupus phenotype could not be defined (*n* = 7), unless the IFN-α concentrations during all visits were below the median (*n* = 1). In total, 24 patients had a high IFN-α lupus phenotype, 33 patients had a low IFN-α lupus phenotype, and 9 patients were undetermined.

### Statistical analysis

Exploratory data analysis and statistical analyses were implemented using R version 4.3.1 (R Core Team, 2022). To analyze the repeatedly measured plasma NfL concentrations as the dependent variable we used a linear mixed-effects model nested within each subject ID with a random intercept. For all models, a generic stepwise model selection procedure was utilized to refine both the random and fixed components of the model, employing the lmerTest package in R. Time-varying covariates included in all models as adjusted predictors were: age, disease duration, plasma creatinine concentrations, and ongoing treatment with prednisolone, hydroxychloroquine, non-antimalarial DMARDs, anti-platelet or anticoagulation therapy, and anti-hypertensives. Each model incorporated either serum IFN-α or the SLEDAI-2 K variables as the separate time-varying predictor of interest to assess with the adjusted covariates. Prior to analysis, missing data for covariates (antihypertensives *n* = 8, antiplatelet/anticoagulation *n* = 3) were imputed using the missForest package in R, which employs a random forest approach. Additionally, multiple logistic and linear regression models were performed to analyze the associations with cognitive dysfunction and MRI variables, respectively. These models utilized dichotomized high/low IFN-α or moderate-high/low SLEDAI-2 K lupus phenotype groups as independent variables. Age at the time of MRI was included as a covariate in the linear regression model, while disease duration was included as a covariate in both models as a proxy for cumulative disease burden. To avoid collinearity and maintain focus on the hypothesized relationships, other variables were not included as independent covariates in the primary analyses. However, to support model comparability, secondary analyses were performed considering additional covariates when differences in clinical features between lupus phenotypes were observed, which applied to the SLEDAI-2 K but not IFN-α phenotypes. Treatments administered at the time of the cross-sectional study were considered additional covariates in the SLEDAI-2 K lupus phenotype models. IBM SPSS Statistics version 28 was employed for these analyses. The threshold for statistical significance was set at *p* < 0.05.

## Results

### Clinical characteristics of the 66 SLE patients

The clinical characteristics of the 66 SLE patients from the cross-sectional study are presented in Table 1. Most patients displayed low disease activity according to SLEDAI-2 K at their visit and a low cumulative level of organ damage according to the SLICC/ACR-Damage Index. No patients had severe renal damage (chronic kidney disease stage 4 or 5). Two-thirds of the patients had at least mild cognitive impairment in any domain and 28% had moderate to severe impairment in at least two domains. A history of clinical apparent nervous system involvement attributed to SLE varied between 23 and 62% of the subjects according to the three applied NPSLE attribution models with different stringencies [33]. Seven patients were active smokers, of whom four reported more than 10 cigarettes per day. No patients reported an alcohol consumption exceeding the Swedish recommended weekly limits and 33% abstained from alcohol.

**Table 1:** Clinical characteristics of the 66 SLE patients at their last visit (time of cross-sectional study)

Age at SLE diagnosis, years, median [IQR] (range)	24 [19–32) (8–42)
Age at cross-sectional study, median [IQR] (range)	37.5 [28.5–44] (18–52)
Disease duration, years, median [IQR] (range)	10.5 [6–18] (0–32)
SLICC SLE Classification Criteria, n, median [IQR] (range)	8 [7–9] (4–13)
Acute cutaneous lupus erythematosus, n (%)	50 (76%)
Alopecia, n (%)	20 (30%)
ANA, n (%)	66 (100%)
Antiphospholipid antibodies, n (%)	21 (32%)
Arthritis, n (%)	54 (82%)
Chronic cutaneous lupus erythematosus, n (%)	18 (27%)
Renal involvement, n (%)	31 (47%)
Serositis, n (%)	27 (41%)
SLICC/ACR-Damage Index ≥ 1 point, n (%)	24 (36%)
History of NPSLE according to ACR / SLICC A / SLICC B model, n (%)	41 (62%) / 15 (23%) / 21 (32%)
SLEDAI-2 K ≥ 4, n (%)	18 (27%)
Hydroxychloroquine ongoing, n (%)	51 (77%)
Any non-antimalarial DMARD ongoing, n (%)	42 (64%)
Azathioprine, n (%)	22 (33%)
Mycophenolate mofetil, n (%)	16 (24%)
Cyclosporine, n (%)	0
Cyclophosphamide, n (%)*	1 (1.5%)
Belimumab, n (%)	8 (12%)
Rituximab, n (%)	1 (1.5%)
Methotrexate, n (%)	1 (1.5%)
Intravenous immunoglobulin, n (%)	2 (3%)
Prednisolone ongoing, n (%)	54 (82%)
Daily dose, mg, median [IQR] (range)	5 (2-5.25) [0–25]
Antihypertensives ongoing, n (%)	22 (33%)
Smoking, current / former / never	7 (11%) / 17 (26%) / 41 (63%)
Moderate to severe cognitive impairment in ≥ 1/12 domains, n (%)	33 (51%)
Moderate to severe cognitive impairment in ≥ 2/12 domains, n (%)	18 (28%)
Mild to severe cognitive impairment in ≥ 1/12 domains, n (%)	43 (66%)
Mild to severe cognitive impairment in ≥ 2/12 domains, n (%)	29 (45%)
IFN-α lupus phenotype high / low (9 patients undetermined and excluded), n (%)	24 (42%) / 33 (58%)
SLEDAI-2 K lupus phenotype moderate-high / low, n (%)	31 (47%) / 35 (53%)
Visits per patient with registered SLEDAI-2 K in the Lund Lupus Cohort database (of total 576 visits - maximum one per year), median [IQR] (range)	8 [5–12] (1–27)

**Abbreviations**: SLE: systemic lupus erythematosus. IQR: interquartile range. SLICC: Systemic Lupus Erythematosus International Collaborating Clinics. N: number. ANA: Antinuclear antibody. ACR: American College of Rheumatology. NPSLE: neuropsychiatric systemic lupus erythematosus. SLEDAI-2 K: SLE Disease Activity Index 2000. DMARD: disease-modifying antirheumatic drug. IFN: interferon. *Ongoing cyclophosphamide includes an ongoing treatment protocol

The clinical and laboratory characteristics from the 199 retrospectively selected visits with concomitant blood samples are presented in Table 2. Visits in which stored blood samples were available within a year after SLE diagnosis were included from approximately half of the patients. In the majority of the 199 visits, patients had ongoing treatment with DMARDs and/or prednisolone. SLE was clinically active (SLEDAI-2 K ≥ 4) in 40% of the visits, very active (SLEDAI-2 K ≥ 10) in 14% of the visits, clinically inactive yet immunologically active according to complement consumption or anti-dsDNA-activity in 23% of the visits, and both clinically and immunologically inactive according to SLEDAI-2 K in 33% of the visits. The most prevalent ongoing clinical disease activity according to SLEDAI-2 K during the 199 visits were skin or mucosal involvement, renal involvement, and arthritis. The visits scarcely involved ongoing neurological or psychiatric activity according to SLEDAI-2 K (*n* = 3).

**Table 2:** Clinical and laboratory data from the retrospectively selected 199 visits

Number of visits & samples per patient, median [IQR] (range)	3 [3–4] (1–4)
First sample within 1 year from SLE diagnosis, n (%)	31 (47%)
Age at sample collection, years, median (range)	31 (13–51)
Disease duration, years, median [IQR] (range)	6 [2–11] (0–30)
SLEDAI-2 K, median [IQR] (range)	2 [0–6] (0–26)
SLEDAI-2 K ≥ 10, n (%)	28 (14%)
SLEDAI-2 K ≥ 4, n (%)	80 (40%)
SLEDAI-2 K zero, n (%)	66 (33%)
SLEDAI-2 K anti-dsDNA and/or low complement only, n (%)	46 (23%)
SLEDAI-2 K seizure/psychosis/OBS/VD/CN/headache/stroke, n	0/0/1/1/0/1/0
SLEDAI-2 K skin or mucosal involvement, n (%)	43 (22%)
SLEDAI-2 K renal involvement (*n* = 192), n (%)	27 (14%)
SLEDAI-2 K arthritis, n (%)	18 (9%)
Hydroxychloroquine ongoing, n (%)	134 (67%)
Any non-antimalarial DMARD ongoing, n (%)	118 (59%)
Azathioprine, n (%)	55 (28%)
Mycophenolate mofetil, n (%)	39 (20%)
Cyclosporine, n (%)	15 (7.5%)
Cyclophosphamide, n (%)	7 (3.5%)
Belimumab, n (%)	7 (3.5%)
Rituximab, n (%)	6 (3.0%)
Methotrexate, n (%)	5 (2.5%)
Intravenous immunoglobulin, n (%)	3 (1.5%)
Prednisolone ongoing, n (%)	143 (72%)
Daily dose (missing *n* = 13), mg, median [IQR] (range)	5.0 [0–10] (0–60)
Antihypertensives ongoing (*n* = 191), n (%)	31 (16%)
Antiplatelet or anticoagulative treatment ongoing (*n* = 196), n (%)	48 (24%)
Plasma creatinine concentration, µmol/L, median (range)	61 (32–108)
Plasma NfL concentration (*n* = 196), pg/mL, median (range)	5.8 (1.2–139.0)
Serum IFN-α concentration, fg/mL, median (range)	122.4 (< 10-14664)

**Abbreviations**: IQR: interquartile range. SLE: systemic lupus erythematosus. N: number. SLEDAI-2 K: SLE Disease Activity Index 2000. Anti-dsDNA: anti-double stranded DNA antibodies. OBS: organic brain syndrome. VD: visual disturbance. CN: cranial neuropathy. DMARD: disease-modifying antirheumatic drug. SLEDAI-2 K: SLE Disease Activity Index 2000. NfL: neurofilament light. IFN: interferon

Both high and low IFN-α lupus phenotype patients had relative increases of IFN-α concentrations during active lupus compared with inactive lupus according to SLEDAI-2 K, however, high IFN-α lupus phenotype patients had higher absolute increases.

At the time of the cross-sectional study, no significant differences in age at study, disease duration, age at diagnosis, alcohol consumption, prednisolone daily dose, fatigue scores, depressive scores, anxiety score, nor average annual SLEDAI-2 K scores (based on all available visits) were seen in high versus low IFN-α lupus phenotype patients. At the time of the cross-sectional study, the frequency of treatment with ongoing DMARDs, prednisolone, antihypertensives, smoking history, history of clinical nervous system involvement according to the three NPSLE models, organ damage according to the SDI, or the SLICC SLE classification criteria (including the presence of antiphospholipid antibodies) did not significantly differ in patients with a high versus low IFN-α lupus phenotype, except leukopenia (75% vs. 42%, *p* = 0.014) and chronic cutaneous lupus (42% vs. 15%, *p* = 0.025), which was more present in high IFN-α lupus phenotype patients.

In moderate-high compared with low SLEDAI-2 K phenotype patients, at the time of the cross-sectional study, average annual SLEDAI-2 K score (5.8 versus 1.3, *p* = 7.0*10^− 7^), ongoing prednisolone daily dose (median 5 versus 4 mg, *p* = 0.014), frequency of ongoing DMARD treatment (77% versus 51%, *p* = 0.025), prednisolone (97% versus 69%, *p* = 0.003) and antihypertensives (48% versus 20%, *p* = 0.015) were higher. No significant differences in age at study, disease duration, age at diagnosis, alcohol consumption, fatigue scores, depressive scores, anxiety score, were observed in moderate-high versus low SLEDAI-2 K phenotype patients. Moreover, NPSLE and organ damage according to the SDI was not more common in moderate-high versus low SLEDAI-2 K phenotype patients.

### Higher disease activity is associated with increased ongoing neuronal damage

Higher SLEDAI-2 K scores were associated with higher plasma NfL concentrations when adjusting for ongoing treatment, age, disease duration, and plasma creatinine concentrations (Table 3). A similar trend was seen for higher serum IFN-α concentrations, however not statistically significant. Visits with SLEDAI-2 K points for anti-dsDNA and/or low complement only were not associated with higher NfL levels.

We assessed the relationship between plasma NfL and clinical disease activity in specific organ systems if at least 10 visits included any point in the specific SLEDAI-2 K item/category. Visits with active renal involvement were associated with higher plasma NfL concentrations.

**Fig. 2:**
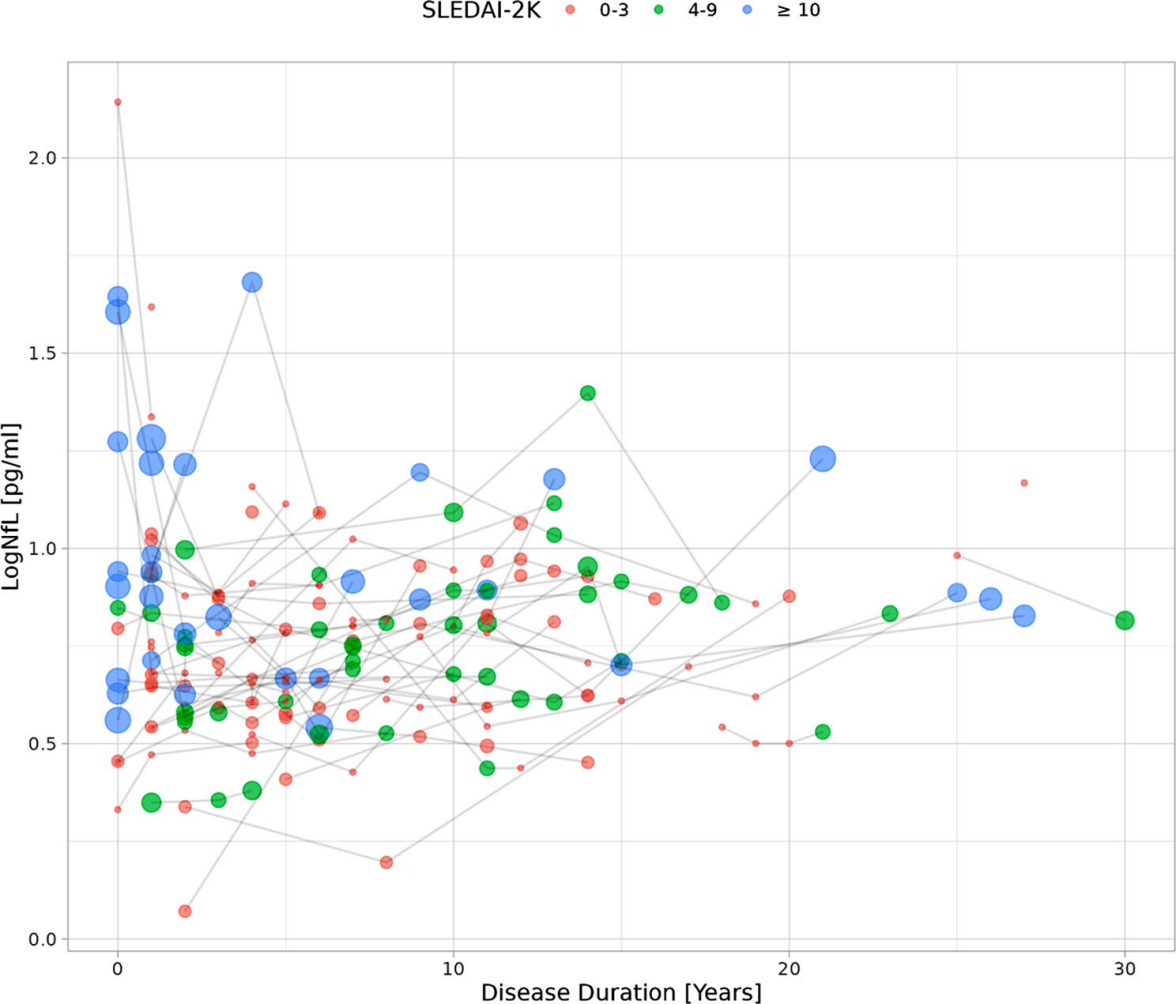
Plasma NfL concentrations over time. The interconnected dots represent the SLEDAI-2 K score from each visit of the 66 patients. Relatively higher disease activity scores and plasma NfL concentrations were observed in visits close to the diagnosis of SLE

Plasma NfL concentrations were particularly high soon after SLE diagnosis in the selected visits, corresponding with high SLEDAI-2 K scores (Fig. 2). Plasma NfL concentrations within two years from diagnosis were significantly higher than 2–5 years (mean LogNfL 0.89 pg/ml; 0.70 pg/ml; *p* = 0.005) or more than 5 years from diagnosis (mean LogNfL 0.89 pg/ml; 0.78 pg/ml; *p* = 0.04).

**Table 3:** Associations between SLEDAI-2 K scores or serum IFN-α concentrations and plasma NfL concentrations

SLEDAI-2 K or IFN-α variables	Estimated fixed effect	SE	T-value	*p*-value
SLEDAI-2 K total	0.015	0.003	5.0	1.5*10^− 6^
SLEDAI-2 K ≥ 10 (*n* = 28) versus < 10 (*n* = 171)	0.24	0.04	5.6	7.2*10^− 8^
SLEDAI-2 K ≥ 4 (*n* = 80) versus < 4 (*n* = 119)	0.095	0.03	2.9	0.0038
Anti-dsDNA and/or low complement only (*n* = 46) versus SLEDAI-2 K = 0 (*n* = 66)	6.3*10^− 3^	0.05	0.13	0.90
SLEDAI-2 K arthritis (*n* = 18 versus *n* = 181)	-0.021	0.06	-0.35	0.72
SLEDAI-2 K renal involvement (*n* = 27 versus *n* = 165)	0.13	0.05	2.6	0.010
SLEDAI-2 K skin or mucosal involvement (*n* = 43 versus *n* = 156)	0.057	0.04	1.4	0.17
LogIFN-α serum concentration	0.047	0.03	1.8	0.081

**Abbreviations**: SLEDAI-2 K: SLE Disease Activity Index 2000. IFN: interferon. Anti-dsDNA: anti-double stranded DNA antibodies. DMARD: disease-modifying antirheumatic drug. SE: Standard error

### The lupus phenotypes with more disease activity over time or with persistently high serum IFN-α levels are associated with structural MRI alterations

High IFN-α phenotype patients exhibited smaller volumes of total grey matter, thalamus, and caudate nucleus compared with low IFN-α phenotype patients (Table 4). Additionally, patients with a moderate-high SLEDAI-2 K lupus phenotype expressed smaller total grey matter volumes, thalamus volumes, alongside with a higher volume of white matter lesions compared with low SLEDAI-2 K phenotype patients (Table 4). The results remained statistically significant when adding ongoing treatment with prednisolone, DMARDs and antihypertensives at the time of MRI to the model (data not shown).

**Table 4:** Associations between MRI abnormalities and lupus phenotypes

MRI volumes [mm^3^/mm^3^]	High versus low IFN-α phenotype		Moderate-high versus low SLEDAI-2 K phenotype	
	**Estimated coefficient (95% CI)**	**P-value**	**Estimated coefficient (95% CI)**	**P-value**
White matter lesion volume	-0.026 (-0.36–0.31)	0.88	0.38 (0.089–0.66)	0.011
Volume of all CSF spaces	3.5*10^− 5^ (-3.5*10^− 5^– 1.1*10^− 4^)	0.32	2.8*10^− 5^ (-3.7*10^− 5^– 9.3*10^− 5^)	0.40
Total cortex volume	-0.0060 (-0.013–0.0013)	0.10	-0.0037 (-0.010–0.0029)	0.26
Total white matter volume	-0.0040 (-0.010–0.0021)	0.19	6.1*10^− 4^ (-0.0049–0.0061)	0.83
Corpus callosum volume	-1.4*10^− 4^ (-3.1*10^− 4^– 4.0*10^− 5^)	0.13	-1.1*10^− 4^ (-2.7*10^− 4^– 4.0*10^− 5^)	0.14
Total grey matter volume	-0.010 (-0.019– -6.5*10^− 4^)	0.036	-0.0088 (-0.017– -3.8*10^− 4^)	0.041
Caudate nucleus volume	-3.3*10^− 4^ (-6.5*10^− 4^– -2.0*10^− 5^)	0.038	-1.8*10^− 4^ (-4.6*10^− 4^– 1.0*10^− 4^)	0.20
Pallidus volume	-5.1*10^− 5^ (-1.8*10^− 4^– 7.6*10^− 5^)	0.42	-6.6 *10^− 5^ (-1.9*10^− 4^– 5. *10^− 5^)	0.29
Putamen volume	-2.8*10^− 4^ (-6.3*10^− 4^ − 6.8*10^− 5^)	0.11	-2.0*10^− 4^ (-5.3*10^− 4^– 1.3*10^− 4^)	0.22
Hippocampus volume	-1.3*10^− 4^ (-3.6*10^− 4^– 1.0*10^− 4^)	0.27	-8.7*10^− 5^ (-3.0*10^− 4^– 1.3*10^− 4^)	0.42
Amygdala volume	-8.8 *10^− 5^ (-2.2*10^− 4^– 4.7*10^− 5^)	0.20	-3.8*10^− 6^ (-1.3*10^− 4^– 1.2*10^− 4^)	0.95
Accumbens volume	-4.3*10^− 5^ (-1.2*10^− 4^ − 2.8*10^− 5^)	0.23	-2.0*10^− 5^ (-8.5 *10^− 5^– 4.5 *10^− 5^)	0.54
Thalamus volume	-5.1*10^− 4^ (-9.4*10^− 4^– -7.4 *10^− 5^)	0.023	-5.3*10^− 4^ (-9.0*10^− 4^– -1.6*10^− 4^)	0.005

Abbreviations: IFN: Interferon. SLEDAI-2 K: SLE Disease Activity Index 2000. CI: Confidence interval. CSF: Cerebrospinal fluid

### The high IFN-α lupus phenotype is associated with a higher degree of cognitive dysfunction

High IFN-α phenotype patients exhibited a higher prevalence of cognitive dysfunction compared with the low IFN-α phenotype patients across multiple domains, with a significantly higher odds of displaying at least mild dysfunction in any domain, moderate-severe dysfunction in any domain, and at least mild dysfunction of processing speed, when accounting for disease duration (Table 5). Additionally, high IFN-α phenotype patients had an increased prevalence of at least mild cognitive dysfunction in at least two domains, however, not significant when accounting for disease duration. No significant differences were observed between moderate-high and low SLEDAI-2 K lupus phenotype patients (data not shown).

**Table 5:** Associations between IFN-α lupus phenotypes and cognitive dysfunction

Cognitive dysfunction in specific domain:	**Degree**	IFN-α lupus phenotype				
		**High**	**Low**	P-value*	**Odds ratio (95% CI)**	P-value**
Any domain	Moderate to severe	70%	39%	0.026	3.5 (1.1–11.1)	0.030
	Mild to severe	91%	49%	0.00087	11.0 (2.2–54.9)	0.004
At least two domains	Moderate to severe	39%	24%	0.23	2.0 (0.61–6.3)	0.26
	Mild to severe	65%	36%	0.034	3.1 (0.99–9.5)	0.053
Neurocognitive Index	Moderate to severe	5%	6%	1.0		
	Mild to severe	9%	6%	1.0		
Composite Memory	Moderate to severe	22%	6%	0.11		
	Mild to severe	30%	15%	0.17		
Complex Attention	Moderate to severe	14%	9%	0.68		
	Mild to severe	14%	13%	1.0		
Cognitive Flexibility	Moderate to severe	9%	9%	1.0		
	Mild to severe	17%	13%	0.71		
Psychomotor Speed	Moderate to severe	4%	3%	1.0		
	Mild to severe	17%	6%	0.22		
Reaction Time	Moderate to severe	22%	18%	0.74		
	Mild to severe	39%	18%	0.13		
Visual Memory	Moderate to severe	22%	6%	0.11		
	Mild to severe	30%	9%	0.073	4.2 (0.95–18.7)	0.058
Processing Speed	Moderate to severe	9%	3%	0.62		
	Mild to severe	26%	6%	0.035	7.9 (1.2–51)	0.029
Executive Function	Moderate to severe	9%	9%	1.0		
	Mild to severe	13%	19%	0.72		
Simple Attention	Moderate to severe	13%	19%	1.0		
	Mild to severe	13%	19%	1.0		
Motor Speed	Moderate to severe	0%	13%	0.52		
	Mild to severe	22%	13%	0.60		
Verbal Memory	Moderate to severe	17%	6%	0.22		
	Mild to severe	26%	15%	0.31		

Abbreviations: IFN: Interferon. OR: Odds ratio. CI: Confidence interval. *Chi-square test or Fischer’s exact test as appropriate. **Logistic regression model with disease duration as a covariate

## Discussion

**Fig. 3:**
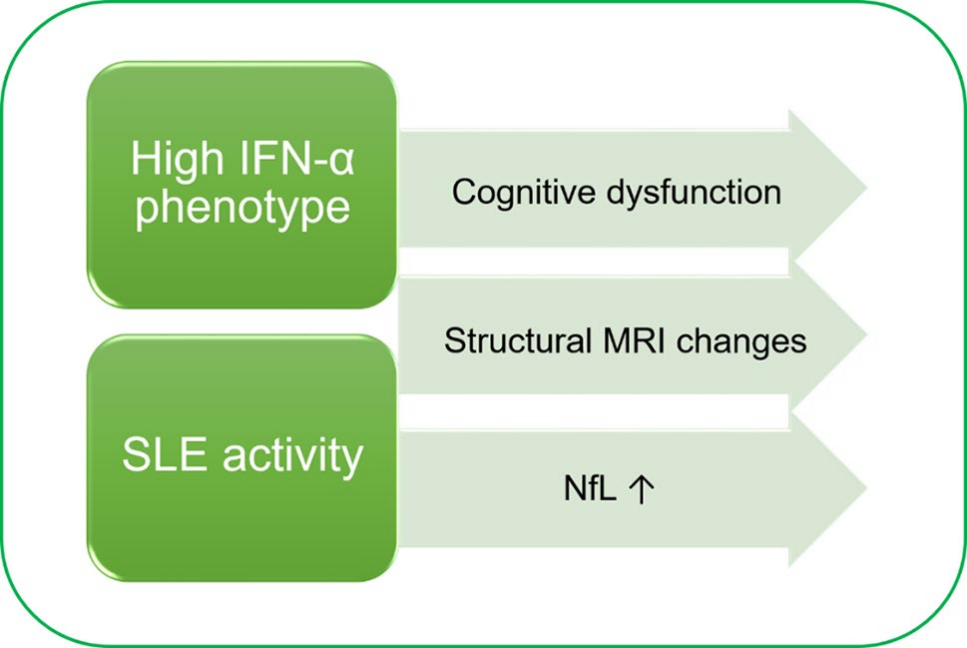
Main findings. This figure illustrates the main findings of this study. Patients with a phenotype of persistently high serum interferon-α levels had a higher degree of cognitive dysfunction. Both patients with a phenotype of moderate-high SLEDAI-2 K and high interferon-α had a higher degree of structural MRI changes. Neuronal damage assessed by plasma neurofilament light levels was more prominent during visits with higher SLE disease activity

In this longitudinal study involving young female SLE patients, we verified distinct associations between the overall SLE disease activity or serum IFN-α levels and neuronal affliction (Fig. 3). Firstly, we demonstrated that higher clinical disease activity was associated with ongoing neuronal damage assessed by increased plasma NfL concentrations during multiple visits without ongoing overt neuropsychiatric involvement, while the association between higher serum IFN-α and plasma NfL did not quite reach significance. The demonstrated association between plasma NfL concentrations and SLEDAI-2 K scores in SLE patients confirms the findings of a recent cross-sectional study on 67 SLE patients [40]. We have previously reported cross-sectional associations between higher NfL levels and factors linked to a higher SLE disease burden, such as organ damage (SDI), low complement C3, anti-dsDNA antibodies, and lupus nephritis [16]. Another recent study confirmed the cross-sectional link between higher NfL concentrations and a higher SLE disease burden including a history of lupus nephritis [30]. In this study we could demonstrate an independent association between increased neuronal damage and ongoing renal involvement, but not with ongoing complement consumption and/or positive dsDNA-antibodies without clinical SLEDAI-2 K points. Future studies may explore whether specific immunological factors involved in renal involvement are particularly linked to increased neuronal damage in SLE, or if this lupus phenotype is more vulnerable due to greater disease severity in general. Our findings strengthen our hypothesis that overall SLE disease activity affects the nervous system even in the absence of overt NPSLE, underscoring the importance of reducing SLE disease activity to mitigate the negative effects on the nervous system related to the disease process. Importantly, this study was not designed to evaluate the value of plasma NfL in acute symptomatic NPSLE diagnostics, but rather investigate mechanisms of the chronic disease processes. The observed higher NfL concentrations concomitant with high disease activity near SLE-onset, prior to the establishment of accelerated arteriosclerosis generated by years of inflammatory burden, provides additional support of direct neurotoxic mechanisms of systemic disease activity through yet undetermined mechanisms. A decline in NfL concentrations over time may indicate the impact of settlements on treatment regimens reducing overall SLE disease activity, in line with two studies demonstrating declines in NfL concentrations after initiating immunotherapy in patients with active NPLSE [22, 25]. Subtle neuronal affliction may indeed manifest early in the disease course much like the instance of overt NPSLE which tends to present early in the disease course [41]. Based on our findings, it would be interesting to investigate plasma NfL as a secondary outcome in clinical trials.

Secondly, we showed that both high IFN-α and moderate-high SLEDAI-2 K patients had smaller volumes of total grey matter, and notably in the subcortical structures: thalamus and caudate nucleus (Fig. 3). Previous studies have revealed total grey matter atrophy in SLE patients compared with healthy controls, and most pronounced in subcortical grey matter areas such as the thalamus, putamen and caudate nucleus, areas associated with normal cognitive function [12, 42, 43]. Our findings may suggest a subclinical neurodegenerative process as a part of overall SLE disease activity in certain individuals. In addition, we demonstrated a larger burden of white matter lesions in the individuals with a higher degree of cumulative clinical disease activity. This effect was seen despite a study design excluding participants older than 55 to minimize age-related MRI abnormalities such as non-specific white matter lesions. This finding could signify a higher degree of microangiopathy resulting in compromised integrity of the white matter in this lupus phenotype [44]. The increased white matter lesion burden may be secondary to the inflammatory burden, possibly attributed to factors associated with reduced endothelial health [45]. Diffusion tensor imaging studies have revealed that compromised white matter tissue microstructure, assessed by increased diffusivity, may start shortly after SLE diagnosis, regardless of clinical neuropsychiatric involvement [46]. Further studies involving neuroimaging at disease onset and at repeated assessments should explore whether the alterations observed in our study occur early in the course of the disease, and whether better control of disease activity may prevent the adverse outcomes [31].

Thirdly, we demonstrated that the lupus phenotype of continuously high serum IFN-α levels regardless of clinical activity was associated with a higher degree of cognitive dysfunction (Fig. 3). These findings may partly be due to the direct effects of type 1 interferons on the brain. Patients undergoing IFN-α therapy for other disorders frequently develop cognitive impairment, and type 1 IFN-signalling in the CNS has been linked to cognitive impairment in various disorders, such as the type 1 interferonopathies, HIV-associated neurocognitive disorder, post-COVID-19 syndrome, and age-induced cognitive decline [19, 20, 47–50]. Cognitive impairment may be attributed to IFN-induced glutamate alterations in areas such as the basal ganglia and dorsal anterior cingulate cortex [51, 52]. Other mechanisms may be IFN-α-mediated neurotoxicity through the GluN2A subunit of the *N*-methyl-*D*-aspartate receptor (NR2) or through IFN-α-activated receptor signalling within cerebral endothelial cells [18, 53]. Our demonstrated association between IFN-α and cognitive dysfunction may be influenced by various factors. One plausible explanation is that IFN-α plays a role in the overall SLE disease process, including endothelial activation, the production of immune complexes and autoantibodies, of which some have an affinity for neuronal structures such as anti-NR2 antibodies, contributing to cognitive impairment [54, 55]. Although no significant differences were observed between the IFN-α lupus phenotype groups regarding glucocorticoid use and dose, DMARD use, anxiety and depressive scores, or a history of antiphospholipid antibodies, these and other factors may still subtly influence the demonstrated association with cognitive outcomes, albeit the lack of differences of these clinical characteristics between the two groups supports model comparability. Given that cognitive dysfunction is a frequent and major issue in SLE, we advocate for additional research investigating the potential cerebral effects of type 1 interferons in SLE patients. We also advocate for prospective studies on new-onset SLE to determine when in the disease course potential IFN-α-induced cognitive dysfunction occurs, as we have previously demonstrated no significant changes in cognitive function during a 5-year follow-up after the cross-sectional study [37]. Furthermore, we propose the inclusion of neurocognitive outcomes when assessing the therapeutic effects of type-1 interferon blocking agents in future trials.

While this study provides novel insights to the association between disease activity and neuronal affliction through a comprehensive longitudinal evaluation of consecutive SLE patients, several limitations should be considered. These limitations include the patient selection, which may limit the generalizability to a broader population of SLE, particularly older subjects and males. Selection bias is a limitation as eligible patients with a more severe SLE disease may have had more frequent clinical visits, increasing the likelihood to participate in research. Conversely, severe fatigue or cognitive dysfunction may have reduced the likelihood to participate, albeit without plausibly influencing the association between active SLE and the neurological outcome. Methodological limitations, including potential measurement errors, the long-term stability of the measured proteins, and the confounding effects of comorbidities, medications, and other variables associated with increased SLE disease activity may impact the analysis on the predicted outcomes. As stated in the *Methods*, not all potential confounders were adjusted for due to collinearity issues, and stratification, beyond phenotype groups, was avoided to preserve statistical power. Addressing these limitations in future research would give an even more nuanced understanding of our findings. Additionally, future studies may incorporate propensity scores to explore associations between treatment, particularly antimalarials, and neurological outcomes.

## Conclusions

In conclusion, we demonstrated that higher disease activity was associated with increased neuronal damage assessed by plasma NfL levels, and that the lupus phenotypes with more disease activity over time or persistently high serum IFN-α levels were associated with long-term structural and functional effects on the brain according to MRI alterations and cognitive dysfunction upon testing. Systemic disease activity including IFN-α may drive neuronal affliction in SLE patients, also in the absence of overt NP-symptoms. This study suggests that effectively managing disease activity could be beneficial for a better cerebral outcome in SLE patients.

## Data Availability

The datasets generated and analysed during the current study are not publicly available due to the privacy of the participating individuals but the data that support the findings of this study are available from the corresponding author upon reasonable request.

## Abbreviations

SLE: Systemic lupus erythematosus
NPSLE: Neuropsychiatric SLE
MRI: Magnetic resonance imaging
IFN: Interferon
NfL: Neurofilament light
SLEDAI-2K: SLE Disease Activity Index 2000
CSF: Cerebrospinal fluid
SLICC: SLE International Collaborating Clinics
ACR: American College of Rheumatology
SDI: SLICC/ACR-Damage Index
VAS: Visual Analogue Scale 100 mm
HADS: Hospital Anxiety and Depression Scale
MADRS-S: Montgomery-Asberg Depression Rating Scale Self-rating version
CNS-VS: CNS-Vital Signs
WML: White matter lesions
DMARD: Disease-modifying antirheumatic drug
ANA: Antinuclear antibody
Anti-dsDNA: Anti-double stranded DNA antibodies
NR2: GluN2A subunit of the N-methyl-D-aspartate receptor
